# Theophylline impact on weaning in oxygen-dependent infants followed in an outpatient Kangaroo Program

**DOI:** 10.3389/fped.2024.1344291

**Published:** 2024-08-20

**Authors:** Adriana Montealegre-Pomar, Nathalie Charpak, Catalina Lince-Rivera

**Affiliations:** ^1^ Research and Projects Department, Kangaroo Foundation, Bogotá, Colombia; ^2^ Pediatrics Department, Pontifical Javeriana University, Bogotá, Colombia; ^3^ Pediatrics Department, San Ignacio University Hospital, Bogotá, Colombia

**Keywords:** theophylline, infant, low birth weight, Kangaroo-mother care method, therapy, oxygen inhalation, pharmacology

## Abstract

**Background:**

Theophylline was an orally administered xanthine used for treatment of apnea of prematurity and Bronchopulmonary Dysplasia in ambulatory follow-up of Low-Birth-Weight infants (LBWI) with oxygen-dependency in the outpatient Kangaroo Mother Care Program (KMCP). Theophylline's main metabolic product is caffeine; therefore, it was an alternative due to the frequent lack of ambulatory oral caffeine in low and middle-income countries.

**Objective:**

To assess the effectiveness of oral theophylline in decreasing days with oxygen and to describe frequency of adverse related events.

**Methods:**

Quasi-experiment before and after withdrawal of theophylline given systematically to LBWI with ambulatory oxygen in two KMCPs.

**Results:**

729 patients were recruited; period 1: 319 infants when theophylline was given routinely and period 2: 410 infants when theophylline was no longer used. The theophylline cohort had less gestational age, less weight at birth, more days in Neonatal Intensive Care Unit, more days of oxygen-dependency at KMCP admission, and more frequencies of Intrauterine Growth Restriction and apneas. After adjusting with propensity score matching, multiple linear regression showed that nutrition was associated with days of oxygen-dependency, but theophylline treatment not. No differences were found in frequencies of readmissions up to 40 weeks, intraventricular hemorrhage or neurodevelopmental problems. Participants in period 2 had more tachycardia episodes.

**Conclusions:**

We did not find association between oral theophylline treatment and the reduction of days with ambulatory oxygen. For the current management of oxygen-dependency in LBW infants, the importance of nutrition based on exclusive breast feeding whenever possible, is the challenge.

## Introduction

According to the Kangaroo Foundation (KF) database, 11,953 oxygen-dependent (OD) low birth weight infants (LBWI) were followed in the ambulatory Kangaroo Mother Care Program (KMCP) in Bogotá, Colombia from 1998 to the first semester of 2021. During this time, the frequency of oxygen dependency decreased significantly, from 43% between 2003 and 2007 to 7% between 2018 and 2021. Patients born with a gestational age (GA) <32 weeks ranged from 41% to 62%. The length of hospital stays varied based on the severity of perinatal issues.

Over the past 24 years, KMCP has been providing care for LBWI with ambulatory oxygen.

Oxygen dependence of this population is especially prevalent in Bogotá, given its altitude (2,600 m above sea level), a situation that has been extensively explored in other international studies (1). A study conducted by the Kangaroo Foundation in 2004 across 12 hospitals in Bogotá found that factors like birth weight, GA, mechanical ventilation, intrauterine growth restriction (IUGR), and the type of healthcare facility (low vs. intermediate-high mortality) were independently associated with bronchopulmonary dysplasia (BPD), which can be severe and sometimes fatal (2). This study followed 194 patients up to 1-year corrected age (CA), where 76% successfully breastfed at term, and growth at 1 year was appropriate; however, 74% of this cohort still required home oxygen at 40 weeks, and at 3 months 23% did. Additionally, about 57% of the cohort were readmitted to the hospital at least once, with 47% of them having respiratory issues (3). A more recent study by the same research group was conducted between 2017 and 2018. Results from 445 patients with a GA ≦ 33 weeks and oxygen dependency at KMCP admission found that 56% had a history of sepsis, and 49% had received invasive ventilation. Multivariate analysis showed that oxygen dependency was associated with low hemoglobin levels at admission, the number of blood transfusions received, and lower GA. Conversely, weight gain and exclusive breastfeeding until oxygen weaning were identified as protective factors (4).

Various therapeutic approaches have been attempted to prevent and treat BPD, including xanthines like Caffeine, Aminophylline, Doxapram, and theophylline, which have been used for over 40 years in the management of premature patients. These medications stimulate respiratory centers, enhance diaphragmatic function, improve lung flexibility, and reduce airway resistance. These effects are crucial in addressing apnea of prematurity and in chronically ventilated newborns who might experience muscular fatigue and atrophy in the diaphragm and skeletal muscles. Improved muscle function contributes to chest wall stability, increasing functional residual capacity, and enabling successful extubation, which has been linked to fewer days requiring oxygen (5–7).

Theophylline, a type of xanthine, is an orally administered medication used for treating respiratory diseases such as asthma and chronic obstructive pulmonary disease (8). However, its use in newborns, particularly for apnea of prematurity and BPD, has challenges due to its erratic absorption and elimination. It has a significantly longer half-life in newborns (approximately 30 h) compared to adults, primarily because of immature cytochrome monooxygenase P450 enzymes (9). Theophylline use has been associated with several adverse effects, including cardiac arrhythmias, nausea, vomiting, headache, diarrhea, irritability, and insomnia, and there is no specific antidote for these effects (10, 11).

In Colombia, generally preterm infants <32 weeks GA receive caffeine for prevention of apneas during hospitalization. Afterwards, this drug is withdrawn, usually 2 weeks before discharge, once the patient was considered not to be at risk of recurrence. Follow up of preterm or LBW infants is done in the ambulatory KMCP according to national guidelines (12). In 2013, the clinical practice guidelines for the management of preterm newborns in Colombia appeared and established the therapeutic use of caffeine for apneas of prematurity (13). Between 1996 and 2017 oral theophylline was systematically used for OD infants. This decision was based on the evidence of reduced oxygen days associated with the use of xanthines given their anti-inflammatory effects and on respiratory automatism and diaphragmatic contractility (7, 8, 14–16). Caffeine is not available in Colombia for outpatient treatment, then theophylline was used as an alternative.

In 2017, when evidence was published about side effects with the prescription of theophylline and there were doubts about the effectiveness in the reduction of oxygen dependency days, routine treatment was discontinued, and recommendations were modified in the new Kangaroo Mother Care (KMC) technical guidelines provided by the Health Ministry (17).

This study aims to evaluate the effectiveness of oral theophylline in reducing the duration of oxygen dependency and to assess the frequency of adverse events associated with this drug.

## Materials and methods

### Study design

This study employed a quasi-experimental design. Outcomes were compared before and after the discontinuation of systematic theophylline treatment for LBWIs receiving ambulatory oxygen.

### Eligibility criteria

All LBWIs with ambulatory oxygen followed in two ambulatory KMCPs from two teaching hospitals, “Hospital Universitario San Ignacio” and “Hospital Infantil San José” in Bogotá, Colombia were included. Infants with a history of seizures, congenital heart disease, or cardiac arrhythmia were excluded from the study.

### Groups of comparison

The first group was followed up between July 25, 2017, and May 31, 2018, period when all patients received theophylline. The second group was followed up between June 01, 2018, and April 20, 2019 and did not receive the drug.

### Outcome measures

Data was collected in the digital medical records of the participating outpatient KMC programmes. This record has a standardized format with data from gestation, perinatal, birth, and follow-up up to 1 year of age.

The study compared several factors, including the number of days requiring oxygen, hospital readmissions, frequency of KMCP consultations, feeding patterns, anthropometric measurements, and the occurrence of tachycardia, gastroesophageal reflux (GER), colic, seizures, and other side effects that could be attributed to oral theophylline treatment at 40 weeks GA and at the time of oxygen weaning.

As a protocol of the outpatient KMCP, these children were evaluated by the pediatrician once they leave the hospitalization on a daily basis until stable weight gain is verified (15–20 g/kg/day), and then weekly until weaning from oxygen. In each evaluation the pediatrician asked parents about events such as apnea, tachycardia, reflux, among others, and emergency room visits for this type of events are also recorded. Patients have a cell phone number which they can use outside consultation hours and which is answered by a pediatrician.

### Sample size

The sample size was determined using STATA 14 software (18). The primary outcome of interest was the days of oxygen dependency during ambulatory KMCP follow-up. According to KMCP data, oxygen-dependent patients required an average of 70 days of oxygen support with standard deviation (SD) of 64 days.

To detect a reduction of 15 days in oxygen requirement with an alpha level of 0.05, a power of 80%, a two-tailed estimation, and accounting for potential follow-up losses of 20%, a sample size of 344 patients per group was calculated. A power calculation was done with the results obtained.

### Intervention

• In the first cohort, LBWI infants with oxygen dependency received oral theophylline (Teolixir®) at a dose of 4 mg/kg/day up to 40 weeks GA. This dose was divided three times a day, and multivitamins were also provided. However, with updated Kangaroo Guidelines in late 2017, theophylline usage was halted for the second cohort, and this transition took place over 4 months.
• All infants in both cohorts received the same outpatient Kangaroo Protocol interventions (17), including:
  ◦ Kangaroo Position (KP): This involves placing the newborn in skin-to-skin contact with the mother 24 h a day, between the mother's breasts and under her clothing. Elastic support is used to maintain this position, ensuring infant body temperature regulation and preventing airway obstruction due to postural changes.
  ◦ Feeding: Infants can be fed at any time while in the Kangaroo Position. Exclusive breastfeeding (EBF) is encouraged, with a strict schedule initially. Weight gain should match intrauterine development (15–20 g/kg/day). If this isn't achieved, supplementation with formula is administered after an assessment to rule out other medical conditions.
  ◦ Follow-up: Patients are monitored daily until they achieve adequate weight gain (15–20 g/kg/day), followed by weekly checks until they reach term (40 weeks GA). This approach is similar to minimal in-hospital care and is termed “minimal ambulatory neonatal care.”
  ◦ Oximetry: Dynamic oximetry with a pulse oximeter is performed regularly to maintain oxygen saturation between 90% and 94% with a healthy heart rate.
  ◦ Parents' education: Parents are educated about managing oxygen at home and provided with instructional materials.
  ◦ Additional Care: The follow-up program includes ophthalmologic screening and early detection of neurological conditions with ultrasound brain sonography. Anthropometric measurements are conducted using Fenton-2013 charts (19).

This comprehensive approach was consistently applied to both cohorts.

### Statistical analysis

The statistical analysis was conducted using Stata 14. Quantitative data, due to its non-normal distribution, was presented as medians along with their interquartile range. Qualitative data was represented through absolute and relative frequencies.

For bivariate analysis of quantitative data, the Mann-Whitney nonparametric test was employed. Qualitative data was analyzed using either the Chi-square test or Fisher's exact test, depending on the specific circumstances.

Taking into account the non-normal distribution of the variable “total days of O2 dependency” we did a power calculation. Using the G*Power 3.1 program (20) for Mann Whitney statistic (median comparison between two different groups), two-tailed difference, Cohen's *d* of 0.27, alpha of 0.05 and sizes of the groups to be compared of 319 and 410 participants respectively, the power of the study was 0.94.

To address any imbalances in baseline variables between the groups, Propensity Score Matching was performed. This process aims to create a balanced comparison group by matching participants with similar characteristics from both cohorts. For propensity score matching, the dependent variable was receiving or not theophylline and the independent variables were those whose baseline characteristics showed a significant difference between the group that received theophylline and the group that did not receive the drug. These were gestational age at birth and at entry to the outpatient Kangaroo Mother Care (KMC) programme, birth weight, history of IUGR, history of apneas, total days in the NICU and total days on oxygen at entry to the outpatient KMC programme. A radius caliper of 0.1 was taken.

## Results

A total of 729 patients were included in the study, with 319 in the theophylline group and 410 in the non-theophylline group.

### Baseline characteristics

• No differences in sociodemographic variables were observed. However, significant clinical differences were noted, with infants in the theophylline group being more fragile. They had lower GA at birth and at entry into the KMCP (*p* < 0.001), lower birth weight (*p* < 0.001) and weight at entry (*p* = 0.026), longer hospital stays (*p* < 0.001), and less use of non-invasive ventilation (*P* = 0.045) (Tables 1, 2).

**Table 1:** Baseline characteristics of the sample population.

Variable		Theophylline group *N* = 319	Non-theophylline group *N* = 410	*P* ^a^
Sociodemographic characteristics				
Father's studies *N* (%)	Elementary	13/299 (4.3)	17/382 (4.4)	0.548
	High School	212/299 (70.9)	284/382 (74.4)	
	Professional or technical	74/299 (24.8)	81/382 (21.2)	
Per capita index. Median (IQR)		0.56 (0.34, 1.06)	0.68 (0.41, 1.28)	0.050
Mother's age. Median (IQR)		31 (25, 36) (16–49)	30 (25, 34) (17–51)	0.169
Clinical characteristics				
Cesarean delivery		255/319 (79.9)	317/410 (77.3)	0.393
Year of recruitment *N* (%)	2017	199/319 (62.4)	59/410 (14.4)	
	2018	119/319 (37.3)	291/410 (71.0)	
	2019	1/319 (0.3)	60/410 (14.6)	
Birth GA (weeks). Median (IQR)		33.0 (30.7, 34.7)	34.4 (32, 35.7)	<0.001*
Birth weight (g). Median (IQR) (Min, max)		1,815 (1,390, 2,151) (605–3,225)	2,000 (1,575, 2,275) (640–2,945)	<0.001*
Birth length (cm). Median (IQR)		43 (40, 46)	44 (41, 46)	<0.001*
Birth head circumference (cm) Median (IQR)		31 (29, 32.5)	31 (30, 32.5)	0.096
GA at KMCP entry (weeks). Median (IQR)		36.0 (34.9, 37.3)	36.9 (35.8, 38.1)	<0.001*
Weight at study recruitment(g)		2,130 (1,940, 2,380)	2,170 (1,980, 2,450)	0.026*
Median (IQR) (Min, Max)		(1,425, 3,470)	(1,090, 5,290)	
Length at study recruitment (cm)		44 (43,46)	44 (43, 46)	0.031*
Median (IQR)		(40–50)	(30–54)	
Head circumference at study recruitment (cm). Median (IQR)		32 (32, 34)	33 (32, 34)	0.026*
Male gender *N* (%)		192/319 (60.2)	235/409 (57.5)	0.458
Apgar score 1 min *N* (%)	1–4	13/280 (4.6)	15/363 (4.1)	0.724
	5–7	113/280 (40.4)	137/363 (37.8)	
	8–10	154/280 (55.0)	211/363 (58.1)	
Apgar score 5 min *N* (%)	1–4	3/280 (1.1)	1 /363 (0.3)	0.233
	5–7	34/280 (12.1)	34/363 (9.4)	
	8–10	243/280 (86.8)	328/363 (90.3)	
NICU hospitalization *N* (%)		213/319 (66.8)	262/410 (63.9)	0.420
Mechanical ventilation (days)		1 (0, 2)	0 (0, 2)	0.119
Median (IQR) (Min, Max)		(0–60)	(0–100)	
Non-invasive ventilation (days)		0 (0, 1)	0 (0, 1)	0.045*
Median (IQR) (Min, Max)		(0–40)	(0–45)	
NICU stay (days)		3 (0, 13)	2 (0, 8)	0.034*
Median (IQR) (Min, Max)		(0–65)	(0–100)	
Total NCU stay (days)		18 (11, 33)	13 (8, 25)	<0.001*
Median (IQR) (Min, Max)		(1–93)	(0–150)	
Days of O2 dependency at KMCP entry		20 (12, 34)	16 (10, 29)	<0.001*
Median (IQR) (Min, Max)		(5–96)	(4–113)	

^a^ Mann-Whitney test for quantitative variables and Chi^2^ or fisher exact test for categorical variables.

*Statistically significant.

**Table 2:** Birth weight and gestational age characteristics, according to groups.

Variable *N* (%)		Theophylline group *N* = 319	Non-theophylline group *N* = 410	*P* Chi^2^
Birth weight	<1,000 g	23 (7.2)	22 (5.4)	0.004*
	≧1,000 < 1,500 g	73 (22.9)	67 (16.3)	
	≧1,500 < 1,800 g	60 (18.8)	58 (14.1)	
	≧1,800 < 2,000 g	46 (14.4)	56 (13.7)	
	≧2,000 g^a^	117 (36.7)	207 (50.5)	
GA	<30	57 (17.9)	40 (9.7)	<0.001*
	>=30 < 32	57 (17.9)	47 (11.5)	
	>=32 < 34	85 (26.6)	82 (20.0)	
	>=34 < 37	108 (33.9)	200 (48.8)	
	>=37	12 (3.7)	41 (10.0)	
IUGR		31 (9.7)	67 (16.3)	0.012*

^a^ The range of birth weights >=2,000 g in the Theophylline group was between 2,000 and 3,225 g and in the non-theophylline group between 2,000 and 2,945 g. Participants were preterm or LBW infants with ambulatory oxygen.

*Statistically significant.

### Follow-up results

Table 3 shows the following results:

• The theophylline group had more cases of BPD when defined as oxygen dependency >28 days and GA 36 weeks (*p* = 0.02) (21). However, when defined as oxygen dependency for >28 days and GA ≧ 40 weeks (22), there was no difference between the groups (*p* = 0.075).
• Infants who received theophylline experienced a higher frequency of apneas during both hospitalization and ambulatory follow-up (*p* = 0.035). Additionally, they reached oxygen weaning at a later chronological age (*p* < 0.001) and had higher weights at 40 weeks (*p* = 0.001). Based on corrected age, there is no difference at oxygen weaning (*p* = 0.112).
• While the feeding pattern at oxygen weaning was similar between the groups, the non-theophylline group had a higher proportion of exclusive breast milk (EBM) at 40 weeks (46% vs. 36%; *p* = 0.002).
• No differences were found in the number of readmissions up to 40 weeks GA, or in the frequency of intraventricular hemorrhage (IVH), retinopathy of prematurity (ROP), or neurodevelopmental problems.
• Regarding potential side effects associated with theophylline treatment, no differences were found in GER or other gastrointestinal disorders. However, more episodes of tachycardia were recorded in the theophylline group (1.3% vs. 0; *p* = 0.038).
• Patients in the theophylline group had a slightly higher percentage of losses (3.5% vs. 1%). However, these losses were generally minor for both cohorts.

**Table 3:** Follow up results until 40 weeks gestational age.

Variable		Theophylline group *N* = 319	Non-theophylline group *N* = 410	*P* Mann-Whitney or Chi^2^
Total days of O2 dependency		54 (40, 81)	47 (35, 66)	<0.001*
Median (IQR)		(12–356)	(14–305)	
Chronological age at O2 weaning		55 (41, 82)	48 (35, 69)	<0.001*
Median (IQR)		(12–356)	(14–306)	
GA at O2 weaning		40.9 (39.1, 43.6)	41.1 (39.7, 42.9)	0.565
Median (IQR)		(35.3–81.6)	(35.9–78.7)	
Weight at O2 weaning		3,400 (2,955, 4,200)	3,390 (2,850, 3,990)	0.112
Median (IQR)		(2,070–8,410)	(1,610–9,320)	
Feeding pattern at O2 weaning *N* (%)	EBF	140/302 (46.4)	181/392 (46.2)	0.988
	BF + F	130/302 (43.0)	168/392 (42.8)	
	F	32/302 (10.6)	43/392 (11.0)	
Weight at 40 weeks. Median (IQR)		3,090 (2,720, 3,370)	2,930 (2,640, 3,300)	0.001*
Length at 40 weeks. Median (IQR)		48 (47, 50)	48 (46, 49)	0.151
HC at 40 weeks. Median (IQR)		35 (34, 36)	35 (34, 36)	
Feeding pattern at 40 weeks *N* (%)	EBF	113/318 (35.5)	188/408 (46.1)	0.002*
	BF + F	191/318 (60.1)	192/408 (47.1)	
	F	14/318 (4.4)	28/408 (6.8)	
IVH *N* (%)	No	307/319 (96.3)	394/410 (96.1)	1.000
	GI	10/319 (3.1)	13/410 (3.2)	
	GII	1/319 (0.3)	2/410 (0.5)	
	GIII	1/319 (0.3)	1/410 (0.2)	
Neurological exam at 40 weeks *N* (%)	Not problems reported	248/293 (84.7)	319/355 (89.9)	0.118
	Hypotonia	37/293 (12.6)	32/355 (9.0)	
	Hypertonia	7/293 (2.4)	4/355 (1.1)	
	Other problems	1/293 (0.3)	0	
At least 1 hospitalization between discharge and 40 weeks *N* (%) (Min, Max)		14/212 (6.6) (0–16)	9/227 (3.96) (0–9)	0.215
Apnea during hospitalization. *N* (%)		43/317 (13.6)	35/404 (8.7)	0.035*
Apnea during ambulatory follow-up 40 weeks. *N* (%)		7/319 (2.2)	2/403 (0.5)	0.049*
GER 40 weeks. *N* (%)		1/319 (0.3)	0	0.442
Feeding intolerance or G/I symptoms 40 weeks. *N* (%)		3/319 (0.9)	0	0.086
Tachycardia-40 weeks. *N* (%)		4/319 (1.3)	0	0.038*
Seizures-40 weeks #. Median (IQR)		(0–1)	0	0.112
BPD (O2 > 28 days). *N* (%)		280/319 (87.8)	350/410 (85.4)	0.346
BPD (O2 > 28 days & GA >= 36 weeks). *N* (%)		298/317 (94.0)	354/406 (87.2)	0.002*
BPD (O2 > 28 days & GA>=40 weeks). *N* (%)		192/319 (60.2)	273/410 (66.6)	0.075
ROP-40 weeks. *N* (%)		25/309 (8.1)	38/370 (10.3)	0.330
% of total attrition in KMCP. *N* (%)		11/319 (3.5)	4/410 (1.0)	0.020*

EBF, exclusive breast feeding; BF + F, breast feeding + formula; F, only formula.

*Statistically significant.

### Multivariate analyses

• Table 4 shows the variables of the propensity score matching and the *p*-values before and after matching, where it can be seen that the balance between the two groups was guaranteed.
• After propensity score matching, linear regression was performed. In this analysis, the number of days requiring oxygen was the dependent variable, and the independent variables were those with a *p* value <0.05 in bivariate analyses. This method allowed us to examine the relationships between various factors and the length of time an infant requires oxygen support. Interactions and confounding variables were evaluated to arrive at the most parsimonious model. Then, model was diagnosed. Table 5 shows the results obtained.
• Finally, multiple linear regression was conducted with total days of oxygen dependency as the outcome variable and theophylline exposure as the primary independent variable. Control variables included the frequency of EBF at oxygen weaning, days of mechanical ventilation, weight at study recruitment, weight gain between study recruitment and oxygen weaning, and weight reached at oxygen weaning (*p* < 0.05 in the model).
• After adjusting for baseline differences through propensity score matching, the analysis showed that EBF could reduce oxygen dependency by 9 days, while each day of mechanical ventilation increased oxygen dependency by 2 days (*p* < 0.001).
• Importantly, no significant association of theophylline administration and total days of oxygen dependency was found in this analysis [682 observations, R2 0.63, 95% CI (−1.01, 4.66) *p* = 0.210].

**Table 4:** Propensity score matching between theophylline and non-theophylline groups.

Variable		Theophylline group	Non-theophylline group	*P*
Gestational age at birth (weeks)	Unmatched	32.60	33.80	<0.0001*
	Matched	32.60	32.80	0.3030
Birth weight (gr)	Unmatched	1,788.50	1,920.70	<0.0001*
	Matched	1,788.50	1,815.90	0.4930
IUGR (%)	Unmatched	0.10	0.17	0.0080*
	Matched	0.10	0.10	0.9150
Days in NICU	Unmatched	8.30	6.85	0.0990
	Matched	8.30	8.10	0.8350
Apneas during hospital stay (%)	Unmatched	0.13	0.09	0.0500
	Matched	0.13	0.11	0.4220
Gestational age at study recruitment (in ambulatory KMC program)	Unmatched	36.20	37.00	<0.0001*
	Matched	36.20	36.30	0.4890
Days of oxygen at study recruitment (in ambulatory KMC program)	Unmatched	25.00	22.00	0.0230*
	Matched	25.00	24.12	0.5380

* Caliper 0.1.

**Table 5:** Multiple linear regression after propensity score matching.

Total days with O2	Coef	Std. Err.	*t*	*p*>*t*	CI 95%
Received theophylline	1.83	1.44	1.27	0.210	(−1.01, 4.66)
Exclusive breastfeeding at O2 weaning	−9.15	1.43	−6.42	<0.001	(−11.95, −6.35)
Days of mechanical ventilation	2.00	0.17	11.95	<0.001	(1.67, 2.33)
Weight at study recruitment in KMC program (gr)	−0.02	0.00	−10.35	<0.001	(−0.02, −0.01)
Weight gain between study recruitment and oxygen weaning (gr/kg/day)	1.08	0.33	3.25	<0.001	(0.43, 1.73)
Weight at O2 weaning	0.03	0.00	20.31	<0.001	(0.02, 0.03)
Weight gain*weight at O2 weaning	−0.00	0.00	−8.32	<0.001	(−0.00, −0.00)
_cons	16.80	6.52	2.58	0.010	(4.00, 29.60)

Number of observations = 682.

*F* (7, 674) = 167.01.

*P* > F = 0.00.

*R*^2 ^= 0.63.

Adj *R*^2 ^= 0.63.

Root MSE = 18.36.

## Discussion

**Figure 1:**
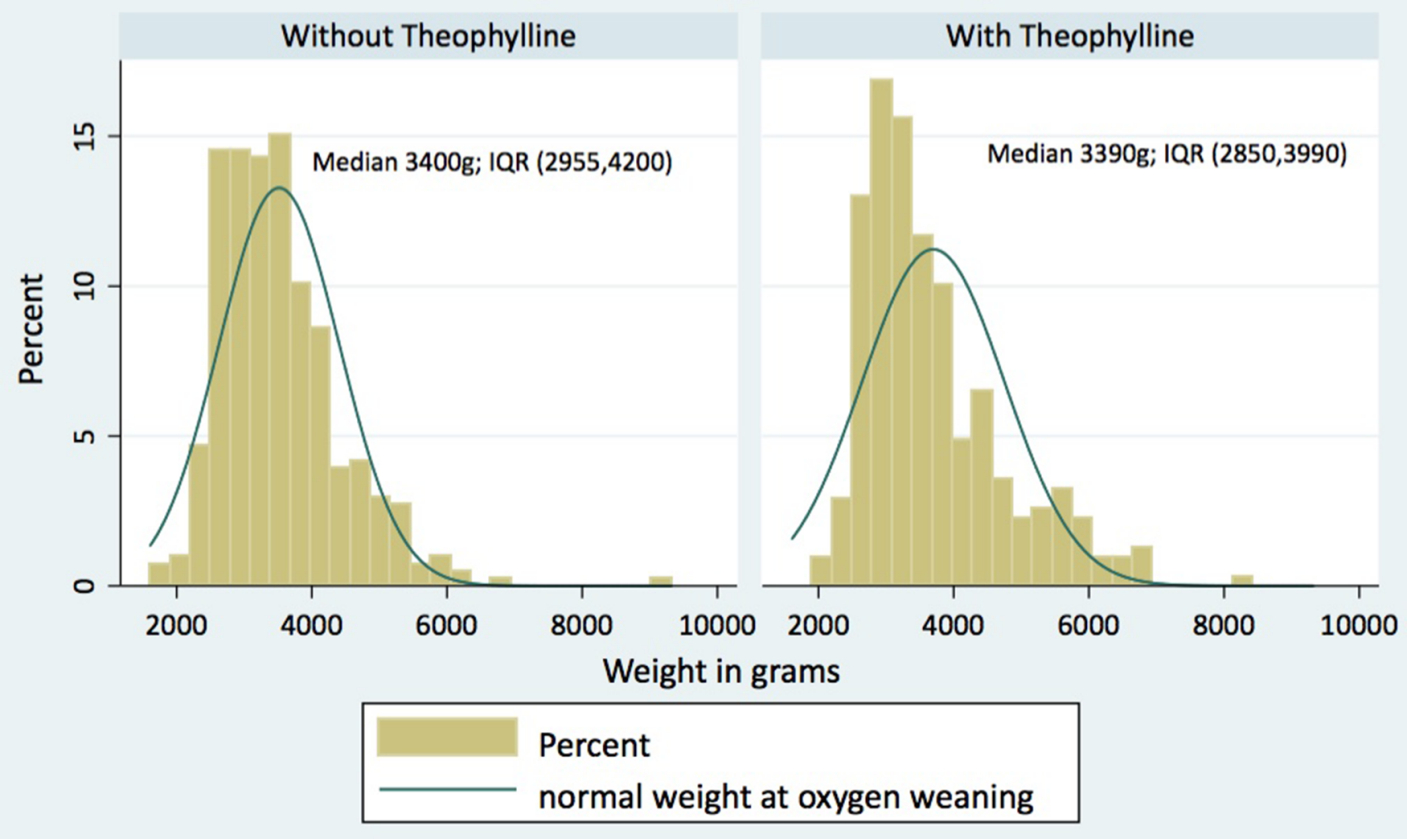
Weight at oxygen weaning.

In this quasi-experiment, we studied the effects of theophylline on LBW infants who required ambulatory oxygen. We compared two groups from two periods, the first, when theophylline was being used routinely, and the second one, when theophylline was no longer used. Children from the first period who received theophylline were more immature, with lower weights and lengths, more frequent apneas, and less opportunity for parenteral nutrition. This led to longer and more complicated neonatal stays, including more days in the NCU. After using propensity score matching for our analysis, the multivariate analysis did not show association between theophylline treatment and the number of days the infants needed oxygen; moreover, infants who received theophylline experienced more episodes of tachycardia. On the other hand, we found that daily weight gain and exclusive breastfeeding were associated with a shorter duration of oxygen dependency. Figure 1 shows that patients in the two groups reached similar medians of weights at oxygen weaning.

We did this study because we believe that in science it is important to evaluate the results of discontinuing an old treatment in the same way as reporting positive results of a new treatment. Physiologically it would appear that theophylline would have effects in reducing oxygen dependency, so it was routinely used for the considerable number of infants on ambulatory oxygen that we followed in Bogotá. However, findings of increased adverse effects in case reports led us to discontinue this drug. In order to obtain better evidence on the effectiveness and safety of theophylline and given the ethical impossibility of conducting a clinical trial, we wanted to conduct this pseudo- experiment before and after the decision of withdrawal.

Bronchopulmonary dysplasia (BPD), the leading cause of oxygen dependency in infants, has significantly evolved in its clinical presentation and definition since earlier studies, such as those by Northway in 1967 (23) and the Kangaroo Foundation in Bogotá in 2004 (2). Advances in neonatal care have reduced lung injury in premature infants, and oxygen dependency is now more often associated with restricted alveolar septation due to antenatal infections (24). Additionally, new respiratory support methods have created controversy regarding the definition of BPD (25–27).

Since 1937, theophylline has been utilized to treat various respiratory diseases, particularly inflammatory conditions like bronchial asthma and chronic obstructive pulmonary disease (8). In newborns, especially preterm infants, it has been employed to manage apnea of prematurity and prevent BPD, thanks to its diuretic and anti-inflammatory properties and its impact on diaphragmatic contractility. This protective effect has also been demonstrated in several studies with caffeine; Zhao et al. in an *in vitro* study found that caffeine reduced NLRP3 inflammasome activation, essential for BPD pathogenesis, and also caspase 1 cleavage leading to decreased IL-1β and IL-18 secretion in THP-1 macrophages (28). The CAP trial showed that caffeine, as compared to placebo, led to earlier successful extubation (from 30.0 to 29.1 weeks GA), discontinuation of positive airway pressure (from 32.0 to 31.0 weeks GA), and removal of oxygen therapy (from 35.1 to 33.6 weeks GA) (14).

Theophylline is metabolized to caffeine, making it available in oral form for outpatient treatment (29). It became a common choice for KMCPs managing LBWIs with ambulatory oxygen (12). However, concerns regarding theophylline's toxicity have arisen over the years. Animal studies have revealed risks like involuntary movements, tachyarrhythmias, ischemic changes, and gastric ulcers (30). In human cases, theophylline overdose has been associated with status epilepticus induction and a potential mortality rate of 10% (9).

On the other hand, caffeine therapy has demonstrated effectiveness with lower risks compared to theophylline. Caffeine is primarily used to manage apnea of prematurity and has shown benefits like reducing BPD and decreasing the risk of death or disability (31, 32). Other advantages are the reduced need for serum level monitoring, the wider therapeutic range, and once-daily administration (33, 34). Recent evidence supports caffeine as the preferred choice for xanthine therapy in newborns.

The growing body of evidence for safer caffeine use has relegated theophylline to a secondary option. Nevertheless, in ambulatory KMCPs in low or middle-income countries, theophylline has continued to be used due to the unavailability of oral caffeine. Recent research has indicated that xanthine use in premature infants is primarily effective for managing apnea of prematurity, with uncertain benefits in reducing oxygen dependency or long-term effects (5). This study was conducted to contribute more evidence on this issue, given the ongoing debate on the role of xanthines in decreasing oxygen dependency.

After adjusting for factors related to improved care over time, such as ventilation practices and nutrition variables, theophylline was not found to reduce oxygen dependency. Instead, exclusive breastfeeding and weight gain were significant factors associated.

These results support the decision to discontinue the systematic use of theophylline in ambulatory programs and emphasize the importance of nutrition, particularly exclusive breastfeeding and monitored weight gain.

Nutrition is recognized as a crucial factor for lung growth and repair, with breast milk providing bioactive components that counteract factors involved in BPD's development, such as oxidative stress and inflammation. The reduction in necrotizing enterocolitis and late sepsis due to breastfeeding may contribute to lower BPD frequency (35, 36).

This study found no significant differences between the two groups in the incidence of BPD at 40 weeks GA, retinopathy of prematurity (ROP), IVH, neurological abnormalities, or re-hospitalizations up to 40 weeks GA. Regarding side effects, there were no variations in gastrointestinal or neurological issues associated with theophylline, but a higher number of tachycardia episodes were recorded, consistent with previous reports.

In the multivariate analysis, factors like weight at admission to ambulatory care, weight gain during follow-up, and exclusive breastfeeding were associated with days of oxygen dependency. On the contrary, we did not find any association with theophylline administration. These findings are in line with the well-established relationship between nutrition and BPD.

This study suggests that exclusive breastfeeding, especially when combined with Kangaroo Mother Care, supports pulmonary tissue repair, nutritional recovery, and growth, resulting in a reduction in days of oxygen dependency. The opening of newborn units in low- and middle-income countries calls for non-aggressive ventilation, early breast milk feeding, humanization, and early discharge to ambulatory KMCPs to improve the survival and quality of life of very premature LBWIs. Current evidence shows that pharmacological therapies have no significant effect in oxygen dependence treatment (16).

The main limitation of this study was the impact of improved perinatal care during the two assessment periods, leading to baseline differences between the groups. In recent years, prenatal care and neonatology practices in Colombia have improved significantly. Premature delivery risks are better detected, birth weights are higher on average, there are fewer preterm births, and in-hospital nutrition for these fragile children is better adapted, including the use of colostrum, early parenteral nutrition with rapid progression, and breast milk (37). This improvement is evident, for example, in the higher frequency of exclusive breastfeeding at discharge in children who did not receive theophylline (46% vs. 36%).

To account for these baseline differences common in observational studies, propensity score matching was performed before multivariate analysis. Results from this study suggest that current neonatal management of oxygen dependency should emphasize optimal nutrition, particularly exclusive breastfeeding, over drug treatment. This approach supports adequate weight gain and pulmonary tissue repair.

## Data Availability

The raw data supporting the conclusions of this article will be made available by the authors, without undue reservation.
